# Antimicrobial resistance patterns and virulence factors of enterococci isolates in hospitalized burn patients

**DOI:** 10.1186/s13104-017-3088-5

**Published:** 2018-01-02

**Authors:** Leili Shokoohizadeh, Alireza Ekrami, Maryam Labibzadeh, Liaqat Ali, Seyed Mohammad Alavi

**Affiliations:** 1 Infectious and Tropical Diseases Research Center, Health Research Institute, Ahvaz Jundishapur University of Medical Sciences, P.O. Box: 61357-15794, Ahvaz, Iran; 2 Department of Medical Laboratory Sciences, Faculty of Para Medicine, Ahvaz Jundishapur University of Medical Sciences, P.O. Box: 61357-15794, Ahvaz, Iran; 3 Infertility Research and Treatment Center of Jahad Daneshgahi, Khuzestan, Ahvaz, Iran; 4 Department of Internal Medicine II, University Hospital Freiburg, Freiburg, Germany; 5 Department of Molecular Medicine, National University of Medical Sciences, Rawalpindi/Islamabad, Pakistan

**Keywords:** *Enterococci*, Vancomycin resistant, Virulence genes, Burn

## Abstract

**Objective:**

The objective of this study was to determine the frequency of the antimicrobial resistance and genes encoding virulence factors of enterococci isolated in hospitalized burn patients in a major burn center in Ahvaz, southwest of Iran. A total of 340 bacterial isolates were collected from the burn center from February 2014 to February 2015. The antimicrobial susceptibility and MIC of vancomycin were determined using the disk diffusion and micro-agar dilution techniques. The genus and species-specific genes, potential virulence genes, and *van*A and *van*B genes were detected by polymerase chain reaction.

**Results:**

According to our results, out of the 340 bacterial isolates, 16.4% (n = 56) were identified as enterococci. Out of the 56 enterococcal isolates, 35 (62.5%) were *Enterococcus faecalis* and 21 (37.5%) were *Enterococcus faecium*. More than 20% (n = 5) of *E. faecium* demonstrated resistance to vancomycin. The *gelE* and *asa* genes were the most prevalent virulence genes in *E. faecalis* (48.5%) and *E. faecium* (43%) isolates. The emergence of vancomycin resistant *E. faecium* strains which have several virulence factors should be considered as a major cause of concern for burn centers. Control and management of infections induced by enterococci should be regarded as highly important in burn patients.

## Introduction

Nosocomial infections are the most common factors in increasing morbidity, mortality, long-term treatment, and cost in hospitalized patients [1, 2]. Burn patients are more at the risk of complications of nosocomial infections due to their weakened immune system [2]. In many countries, particularly developing countries including Iran, burn infections are one of the most serious issues in the field of health [3]. Organisms associated with nosocomial infections in burn patients include the patient’s own normal flora organisms and the hospital environment or the staff. Bacterial pathogens are the most common cause of burn infections such as wounds in burn patients. In addition to wound infections, bacterial pneumonia and bloodstream infections are also common causes of death in burn patients [4].

Among Gram-positive and Gram-negative bacteria, *Staphylococcus aureus* and *Pseudomonas aeruginosa* are considered as the most important factors causing burn wound infections [5]. According to reports published in recent years, enterococci have become important factors in acquiring nosocomial infections [6–8]. These Gram-positive bacteria are part of the normal flora of the human digestive system and were considered to be commensal bacteria in the past [6]. Since enterococci have the ability to cause infection in wounds, including burn wounds, and are intrinsically resistant to many antibiotics due to their ability to acquire antibiotic resistance genes, they are considered as one of the most important causes of nosocomial infections. Enterococci are now regarded as the third most common cause of hospital infections. Enterococci can cause urinary tract, intra-abdominal, pelvic, wound, and super infections (including those caused by burns) in patients [6, 9]. The emergence of multidrug-resistant enterococci strains (resistant to two or more classes of antibiotics) or MDR has caused numerous problems for patients in hospitals all over the world [10]. In *Enterococcus* species, *E. faecium* and *E. faecalis* are regarded as important nosocomial bacterial pathogens. The published reports of some countries indicated the prevalence of burn wound infections caused by VRE strains in hospitals, particularly in the intensive care unit (ICU) [11]. Studies on bacteria and burn infections have been conducted in Taleghani Hospital of Ahvaz as the main burn center in Khuzestan Province, southwest of Iran [12, 13]. Only a few studies have been published on the role of enterococci in burn patients in Iran, and given the increasing importance of VRE-related infections, the aim of the present study was to evaluate the frequency of enterococcal infections, virulence factors, and antibiotic resistance patterns in enterococci strains isolated from clinical samples of burn patients hospitalized in one of the largest burn centers in the southwest of Iran.

## Main text

### Methods

The present study was ethically approved by Ahvaz Jundishapur University of Medical Sciences, Institutional Review Board (Code No. 93146).

A total of 340 bacterial strains were isolated from clinical samples of hospitalized burn patients (wound biopsies, blood, and urine) in a burn center in Ahvaz from February 2014 to February 2015. All the enterococci isolates were identified in the genus and species levels by Gram staining, catalase reaction, growth in 6.5% NaCl solution, motility assessment, use of arabinose, bile, and esculin hydrolysis tests [14]. Furthermore, a PCR-based study was conducted using specific primers (*ddl*_*E. faecium*_ and *ddl*_*E. faecalis*_) for all *E. faecium* and *E. faecalis* isolates [15]. Antimicrobial susceptibility test for enterococci isolates was performed against vancomycin (30 μg), teicoplanin (30 μg), gentamicin (120 μg), ampicillin (10 μg), ciprofloxacin (5 μg), tetracycline (30 μg), chloramphenicol (30 μg), and linezolid (30 μg) (Mast, UK) by the disk diffusion technique. Vancomycin minimum inhibitory concentration (MICs) was detected by micro-agar dilution method based on the CLSI guidelines [17]. DNAs from different enterococcal isolates were extracted using an appropriate DNA Extraction Kit (Cinagene, Iran). Specific primers of *ddl*_*E. faecium*_, *ddl*_*E. faecalis*_ vancomycin resistant genes (*van*A and *van*B), and virulence genes including hyaluronidase (*hyl*), enterococci surface protein (*esp*), haemolysin activator (*cyl*), gelatinase (*gelE*), and aggregation substance *asaI* (Metaboin, Germany) were used for PCR amplification as described previously [14, 16, 18].

### Results and discussion

#### Prevalence of enterococci isolates

Enterococci were isolated from 16.4% (n = 56) of clinical specimens of burn hospitalized patients. The frequency of enterococci in wound, blood, and urine specimens were 37.5% (n = 21), 30.3% (n = 17), and 35% (n = 18), respectively. Among the 56 enterococci strains, 62.5% (n = 35) were identified as *E. faecalis* and 37.5% (n = 21) were identified as *E. faecium.* Table 1 shows the comparison of the frequency distribution of *E. faecium* and *E. faecalis* in different clinical specimens. Among *Enterococcus* species, *E. faecalis* was identified as the major cause of enterococcal infections. *E. faecalis* caused enterococcal infections approximately ten times more than other *Enterococcus* species [19]. *E. faecalis* has more virulence factors than *E. faecium*; consequently, it has higher pathogenicity [20]. Nevertheless, in recent years a microbial shift from *E. faecium* to *E. faecalis* has been observed which could be due to the emergence of MDR strains resistant to vancomycin (VRE) species in hospitals [19, 21]. One of the influential factors in increasing nosocomial infections resulted from enterococci is the emergence of VREs in hospitals. According to the studies conducted in Iran, nosocomial infections caused by enterococci, particularly the resistant strains are highly prevalent [10, 21]. In 2001, the National Nosocomial Infection Surveillance System (NNISS) reported that the frequency of burn wound infections caused by *Enterococcus* was 11% [2] which is close to the results of the current study (16%). There have been few studies investigating the existence of infection caused by enterococci in burn patients. Our results showed an increase in the contribution of *E. faecium* to infections which is consistent with our results on the reported frequency ratio of *E. faecium to E. faecalis* in Tehran’s hospitals in 2014. Although these results are different from other results reported in Iran and other countries, more than 80% of burn wound infections caused by *Enterococcus* was due to *E. faecalis* [6, 22–24].

**Table 1:** Frequency distribution of *E. faecalis* and *E. faecium* isolates in clinical specimens of burn patients

	Urine	Wound	Blood
*E. faecalis* (n = 35)	26% (9)	37% (13)	37% (13)
*E. faecium* (n = 21)	33% (7)	47% (10)	19% (4)

#### Antimicrobial resistance patterns

**Fig. 1:**
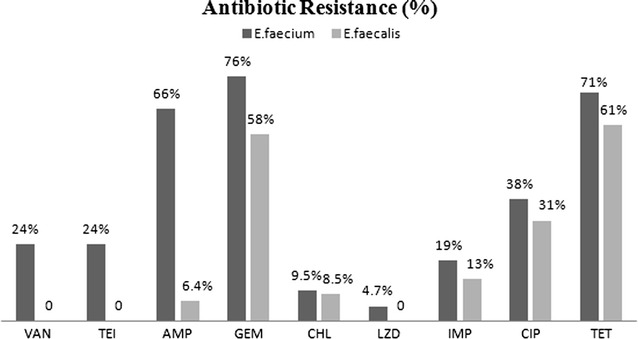
The comparison of antibiotic resistance (%) in *E. faecium* and *E. faecalis* isolates in burn patients

Figure 1 presents the comparison of the results of antimicrobial susceptibility analysis of *E. faecium* and *E. faecalis*. More than 20% (n = 5) of *E. faecium* exhibited resistance to vancomycin and teicoplanin. This can be the reason for the increased presence of *E. faecium* which is resistant to vancomycin and can survive in the hospital environment. *E. faecium* strains exhibited higher levels of resistance to antibiotics. Higher levels of resistance to tetracycline and ampicillin were observed in 71% (n = 15) and 66% (n = 14) of *E. faecium* isolates, respectively. According to the findings, this can be an alarm for burn patients owing to the fact that more than 50% of *E. faecium* strains and 100% of VRE strains were resistant to ampicillin and gentamicin. No resistance to linezolid and vancomycin was observed in *E. faecalis* isolates. The most frequent resistance pattern was simultaneous resistance to ampicillin/tetracycline/gentamicin (AMP/TET/GEM) in 43% of *E*. *faecium* and tetracylin/gentamicin (TET/GEM) in 46% of *E. faecalis* isolates. All vancomycin resistant *E. faecium* (VRE*fm*) possessed *van*A gene, while no *van*B gene was observed in VRE*fm* strains. On the other hand, all VRE strains which possessed *van*A gene showed resistance both to vancomycin and teicoplanin. MIC values for vancomycin ranged from 32 to 1024 µg/ml. In addition, there was no correlation between the presence of vancomycin resistance, virulence factors, and the clinical samples. So far, no documented results for the presence of VRE in burn patients have been published in Iran which may be attributed to the lack of expensive and purposive studies on enterococci in the burn units. However, some countries have reported VREs in burn units [25–28]. According to the results of this study and similar studies, linezolid was identified as the most effective antibiotic against enterococcal infections [10, 22, 23].

#### Distribution of virulence genes

Table 2 presents the frequency and distribution of virulence genes among clinical specimens. The *gelE* and *asa* genes were the most frequent virulence genes in *E. faecalis* (48.5%) and *E. faecium* (43%) isolates, respectively. This is consistent with the results reporting *gel*E as the most frequent virulence factor in *E. faecalis* strains [23, 25, 29], while some reports have indicated the absence or low rate of *gel*E gene in enterococcal isolates [25, 30, 31]. Some reports have indicated the absence or low rate of *asa* gene in *E. faecium* strains [31, 32]. The *cyl* gene was not detected in any *E. faecium* isolates. The *hyl* gene was found in 1.7% (n = 1) of *E. faecium* isolates. A total of 68.5% (n = 24) of *E. faecalis* isolates and 42.8% (n = 9) of *E. faecium* isolates were positive for more than one virulence gene. There was no relationship between virulence genes and clinical specimens.

**Table 2:** Frequency distribution of virulence gene in *E. faecalis* and *E. faecium* isolates

	*esp*	*gel*	*asa*	*hyl*	*cyl*
	*E. faecium/E. faecalis*	*E. faecium/E. faecalis*	*E. faecium/E. faecalis*	*E. faecium/E. faecalis*	*E. faecium/E. faecalis*
Blood	4.7% (1): 16% (5)	0%: 26% (9)	14% (3): 8.5% (3)	0%: 17% (6)	0%: 0%
Urine	9.5% (2): 13% (4)	14% (3): 6% (2)	14% (3): 20% (7)	0%: 8.5% (3)	0: 2.8% (1)
Wound	4.7% (1): 8.5% (3)	19% (4): 17% (6)	14% (3): 20% (7)	4.7% (1): 6% (2)	0: 2.8% (1)
Total	19% (4): 34% (12)	33% (7): 48.5% (17)	43% (9): 48.5% (17)	4.7% (1): 31% (11)	0: 5.6% (2)

In conclusion, based on the results of the current study, it was demonstrated that the detection of enterococci in clinical samples of burn patients is highly important. In addition, the presence of *E. faecium* strains resistant to vancomycin with several virulence factors can be a source of alarm for burn centers. Considering the possible transmission of antibiotic resistance genes among enterococci, and staphylococci, the control and management of infections caused by enterococci as well as the appropriate administration of antibiotics in burn patients can be highly effective in treating nosocomial infections in burn centers.

### Limitations

In this study, no significant differences exist between the presence of virulence genes and resistance to vancomycin. One of the reasons for this result may be the low number of vancomycin resistant strains. Therefore, more samples must be collected from hospitals in general and from burn centers in particular.

## Abbreviations

MIC: minimum inhibition concentration
*E. faecium*: *Enterococcus faecium*
*E. faecalis*: *Enterococcus faecalis*
MDR: multi-drug resistant
VRE: vancomycin resistant enterococci
PCR: polymerase chain reaction
*hyl*: hyaluronidase
*esp*: enterococci surface protein
*cyl*: haemolysin activator
*gle*E: gelatinase
capD: capsular polysaccharides biosynthesis protein
*asaI*: aggregation substance
VAN: vancomycin
TEI: teicoplanin
AMP: ampicillin
GEM: gentamicin
CHL: chloramphenicol
LZD: linezolid
IMP: imipenem
CIP: ciprofloxacin
TET: tetracycline
